# An NCI Micro-credentialing Model for Onboarding and Training Clinical Research Professionals in a Lean Fiscal Environment

**DOI:** 10.1007/s43441-026-00941-z

**Published:** 2026-03-12

**Authors:** Barbara Tafuto, Belinda Zhang, Kathleen Black, Ginnette Watkins-Keller, Rahul Mittal, Barbara DeMarco

**Affiliations:** 1 Rutgers University, New Brunswick, USA; 2 New Jersey Alliance for Clinical and Translational Science, New Brunswick, USA; 3 Rutgers Cancer Institute, New Brunswick, USA

**Keywords:** Clinical research coordinator, Micro-credential, Workforce development, Training and education, National Cancer Center, Onboarding

## Abstract

Clinical research professionals are essential to the successful conduct of clinical trials yet training and retention of this workforce remain significant challenges, particularly with constrained budgets and declining indirect cost reimbursements. This study describes the implementation, and evaluation of a micro-credentialing program at an NCI-designated comprehensive cancer center. The CRC badge, developed through a collaboration between Rutgers School of Health Professions, Rutgers Cancer Institute, and the New Jersey Alliance for Clinical and Translational Science, offers self-paced, competency-based training aligned with the Joint Task Force for Clinical Trial Competency framework. Fifty-six clinical research staff were invited to complete the CRC Badge between May 2023 and May 2024. Survey data from the 38 completers (67%) demonstrated substantial self-reported learning gains across regulatory activities, research roles, and data management. Post-course results indicated that the CRC badge helped enhance onboarding efficiency and inspired interest in continued professional development. Administrative feedback confirmed improvements in staff readiness.

## Introduction

Clinical research professionals (CRPs) play an essential role in the successful conduct of clinical trials, ensuring regulatory compliance, ethical oversight, data quality, and operational efficiency [1]. This workforce, which includes study coordinators, clinical trial managers, research nurses, regulatory affairs staff, and data managers, is foundational to the translational research enterprise at academic medical centers (AMCs) [2, 3]. Since some of these salaries are not included as part of any specific grant, the training and support of CRP staff at institutions such as National Cancer Institute (NCI)-designated cancer centers can be subsidized through indirect costs, the portion of federal research funding allocated to support infrastructure and administrative functions [4].

In 2025, the National Institutes of Health (NIH) implemented a policy capping indirect cost recovery at 15%, down from the national average of approximately 27% to 30% [5]. While often labeled as overhead, indirect costs sustain the infrastructure that underpins research compliance, including Institutional Review Board (IRB) operations, informed consent processes, and ongoing professional training [5, 6]. This reduction in funding poses a direct threat to institutions’ ability to adequately train and maintain their CRP workforce. Without sustained investment in training, research institutions risk diminished study quality, delayed timelines, and reduced capacity to initiate new trials [7, 8].

In this constrained funding environment, micro-credentialing, specifically through digital badges, offers a scalable, cost-effective approach to workforce development [9]. These competency-based tools provide targeted training that allows research staff to build essential skills, adapt to new roles, and meet regulatory demands without the time or financial burden of traditional education programs [10, 11].

The objective of this paper is to understand the value of implementing a micro-credentialing badge as a foundational training mechanism for CRPs at an NCI designated cancer center, emphasizing its potential role in sustaining research operations and institutional resilience amid this new environment of reduced indirect cost funding.

## Background

### NJ ACTS CRC Micro-credential Program

Micro-credentialing represents a forward-thinking investment in the clinical research workforce, fostering both individual career growth and institutional resilience. Micro-credentials or badges have emerged as a viable solution to workforce barriers, facilitating more expeditious training of new hires, while providing clear pathways for improved competencies and career advancement. These structured training initiatives have the potential to alleviate staffing issues and enhance CRP workforce resilience [12].

The CRC Badge/Micro-credentialling program, a collaboration between Rutgers School of Health Professions, Rutgers Cancer Institute, and New Jersey Alliance for Clinical and Translational Science (NJ ACTS), was created to solve staffing issues and supplement the Rutgers Cancer Institute’s training infrastructure. Designed to standardize onboarding and professional development, the CRC badge program ensures foundational training and a standardized assessment for CRCs to attain the core competencies required for effective clinical trial management. This approach is aligned with the competencies defined by the Joint Task Force (JTF) for Clinical Research Competencies, covering areas such as scientific concepts, investigational product development, regulatory requirements, project management, and participant safety [13].

The CRC Badge provides multiple advantages to the institution implementing it, including an allowance for more diverse and expanded job applicants. The structured training encourages candidates from diverse experiential and education backgrounds to apply for a CRP role because the standardization of foundational knowledge prior to the center’s specific onboard training, ensures that new hires possess essential competencies before taking the Rutgers Cancer Institute specific training modules. Fostering uniformity of CRC skills, knowledge, and ethical standards in this foundational clinical research training initiative, contributes to a more efficient, inclusive, and capable workforce.

### Rutgers Cancer Institute and Micro-credentialing as a Workforce Strategy

As part of Rutgers Health, the Rutgers Cancer Institute implemented the CRC Badge as a supplemental onboarding tool to strengthen its clinical research workforce. Rutgers Cancer Institute, New Jersey’s only National Cancer Institute designated Comprehensive Cancer Center, manages over 1200 active clinical trials and enrolls approximately 17% of newly diagnosed adult cancer patients and 70% of pediatric patients. This far exceeds the national adult average of under 5% [14, 15]. These data underscore the centrality of clinical trials to Rutgers Cancer Institute’s mission in therapeutic, diagnostic, and preventive oncology.

Rutgers Cancer Institute’s clinical research professionals (CRPs) are responsible for patient recruitment, informed consent, data management, regulatory oversight, and protocol adherence. To support these roles, Rutgers Cancer Institute’s Office of Human Research Services maintains a structured onboarding program lasting three to six months, depending on role complexity. This curriculum provides progressive training in research logistics, oncology-focused procedures, ethics, regulatory requirements, and clinical trial management systems.

Despite its comprehensiveness, variability in new hires’ backgrounds, decentralized team structures, and ongoing competency assessment needs prompted Rutgers Cancer Institute to explore whether a standardized micro-credential could enhance onboarding efficiency and consistency. The CRC Badge was therefore introduced as an interactive, self-paced educational tool to establish core clinical research competencies as part of the onboarding/orientation training that staff receive.

## Methods

This study evaluated the implementation of the NJ ACTS Clinical Research Coordinator (CRC) Level 1 Badge at Rutgers Cancer Institute between May 2023 and May 2024. The purpose was to examine the integration of the badge into training workflows and to assess immediate learning outcomes and early educational impacts among participating clinical research professionals. This study was reviewed by the Rutgers Institutional Review Board and determined to be exempt.

### Staff Participation

Rutgers Cancer Institute leadership invited a total of 56 clinical research staff members to participate in the CRC Badge program. The participants included both newly hired individuals completing the badge as part of their onboarding process and existing staff completing the training for supplemental professional development. Upon enrollment, participants completed an online registration form via REDCap, which collected demographic data and included a self-assessment of core clinical research competencies. These self-assessments were used to establish baseline perceptions of knowledge and confidence in key topic areas.

The CRC Badge, developed by Rutgers Health and NJ ACTS, is a self-directed online training course composed of five sequential modules, which participants were asked to complete over a six-week period. Modules include: (1) Scientific Concepts and Research Design; (2) Ethical and Participant Safety Considerations; (3) Investigational Products Development and Regulation; (4) Clinical Study Operations and Site Management; (5) Data Management and Informatics. The total estimated time to complete the course was approximately 20 h. To progress through each module, learners were required to achieve a minimum score of 90 percent on a post-module quiz, with up to three attempts allowed. Newly hired participants were assigned the badge early in their onboarding timeline to complement Rutgers Cancer Institute’s internal training. Existing staff were provided time within their regular work schedules to complete the badge [12].

Immediately upon completing the course, participants received a follow-up survey via email. This post-course survey was designed to assess the perceived impact of the badge on participants’ knowledge and preparedness for their clinical research responsibilities. Specifically, the survey asked whether participants felt they had learned “no change,” “a little,” or “a lot” in each of the major content areas. It also asked whether the training experience had motivated them to pursue additional education or skill development in clinical research. Survey items were developed collaboratively by Rutgers Cancer Institute administrators and the CRC Badge program team to align with institutional priorities and evaluate the training’s relevance to daily research responsibilities.

### Administration Participation

In addition to surveying participants, the study team distributed surveys to Rutgers Cancer Institute administrative staff at three and six months after course implementation. These surveys were designed to evaluate management satisfaction with the badge program, perceived impact on employee performance, and staff readiness to perform essential clinical trial tasks. Survey items explored perceived changes in research-related competencies including data collection, data entry and management, informed consent, participant enrollment, regulatory documentation, understanding of research roles, and comprehension of research design differences. These evaluations were used to determine whether the badge facilitated more efficient onboarding and supported institutional goals for workforce preparedness.

## Results

### Participant Completion and Demographics

Of the 56 Rutgers Cancer Institute clinical research staff invited to complete the CRC Badge training, 38 participants (67%) completed the course. Demographic data collected through REDCap surveys showed that the majority of participants identified as female (76%), followed by male (18%) and those who preferred not to disclose gender (5%). The largest racial/ethnic group was Asian (45%), followed by White (37%) and Black or African American (11%). Most participants were between the ages of 25–34 (39%) and 35–44 (29%).

In terms of educational background, 55% held a bachelor’s degree, 21% held a doctoral degree, and 18% held a master’s degree. A majority (58%) had a healthcare-related undergraduate major, while 26% reported a science-related major. All participants completed Human Subjects Research training via the CITI Program. Clinical research experience levels varied: 34% of participants reported having 2 to 5 years of experience, while 11% were new to the field. (See Table 1 for full demographic data.)

**Table 1:**
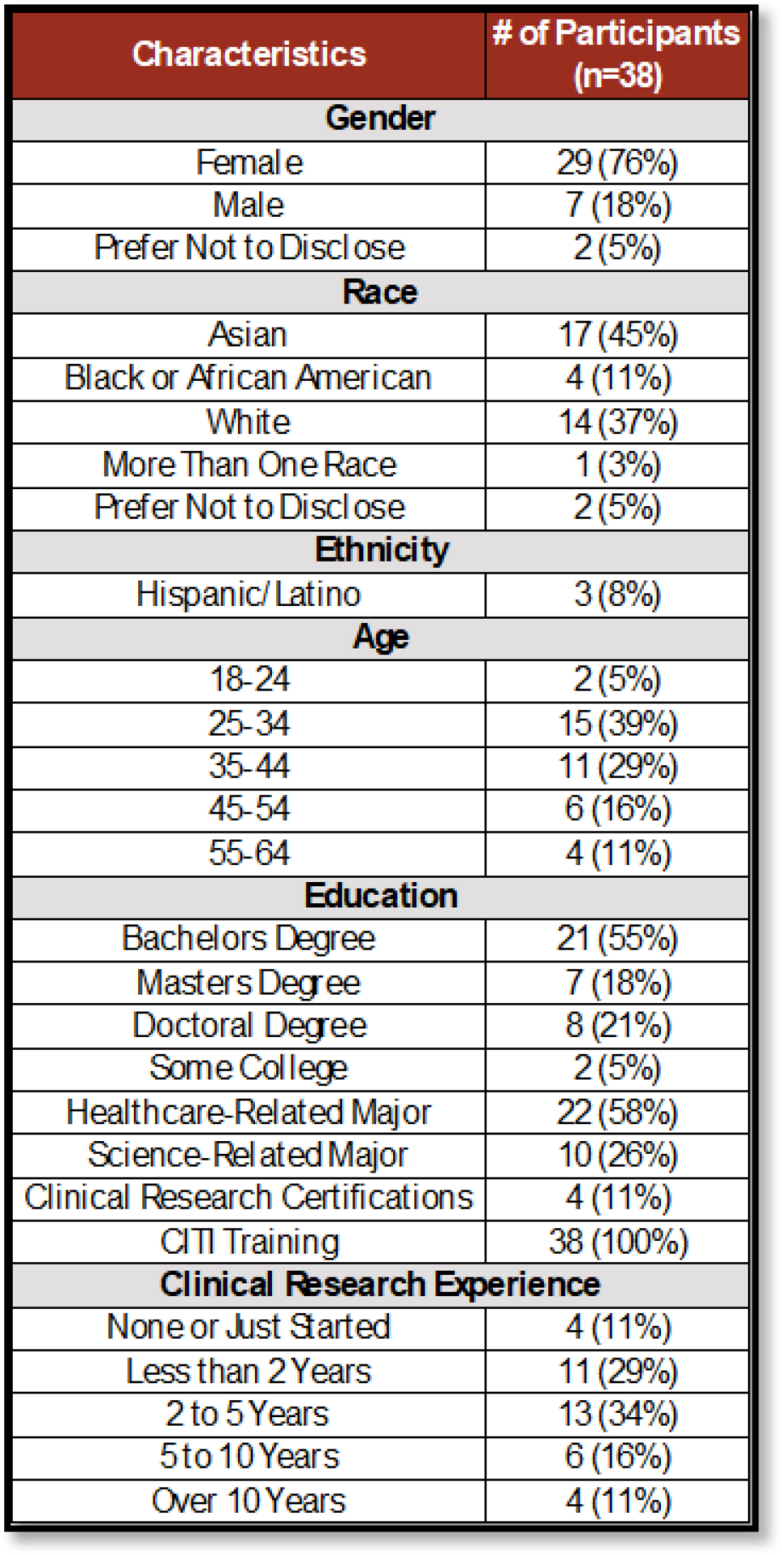
Demographics

### Learning Outcomes (Immediate Post-survey)

**Fig. 1:**
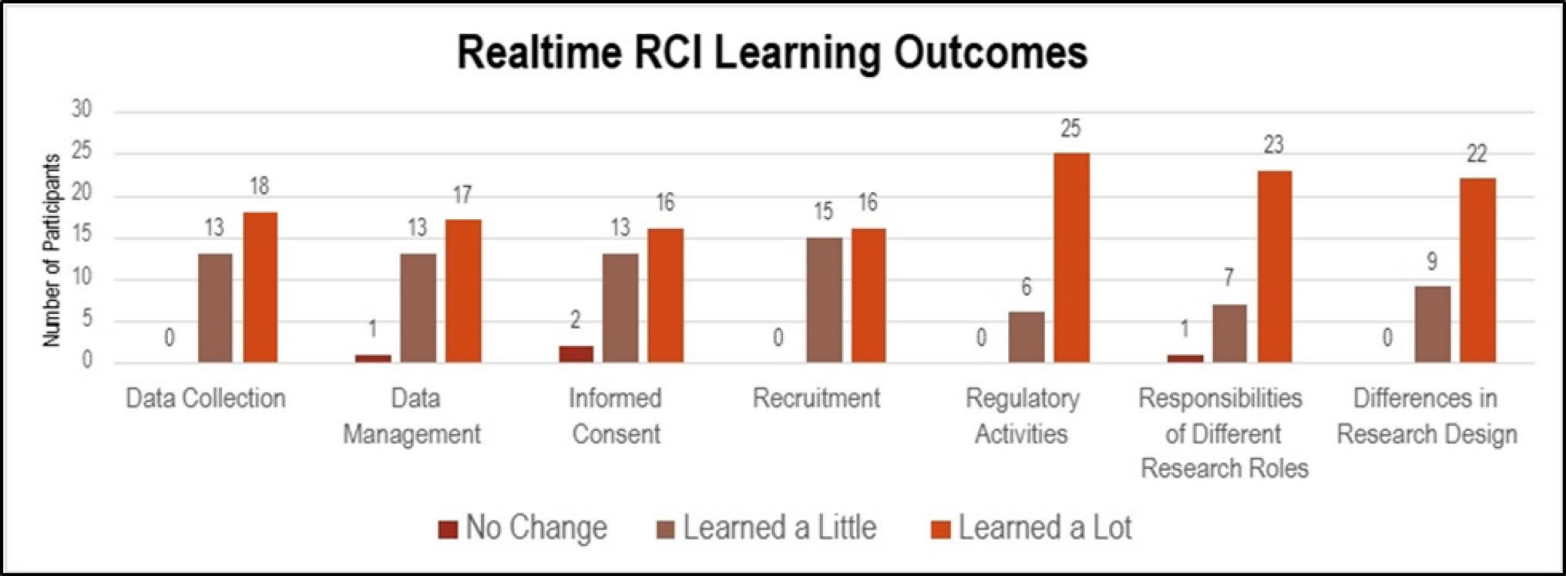
Realtime learning outcomes

All 38 CRC badge completers participated in the immediate post-course survey. Participants were asked to evaluate their perceived knowledge gains in key research domains using the response options “I learned a lot,” “I learned a little,” or “No change.” A substantial proportion of participants reported learning a lot in the areas of Regulatory Activities (n = 25), Responsibilities of Different Research Roles (n = 23), and Differences in Research Design (n = 22). Learning gains were also reported for Data Collection (n = 18), Data Management (n = 17), Informed Consent (n = 16), and Recruitment and Enrollment (n = 16). Only a small number of participants indicated no change in their knowledge across any domain. (See Fig. 1 for a breakdown of perceived learning outcomes.)

### Learning Outcomes by Educational and Experiential Background

**Fig. 2:**
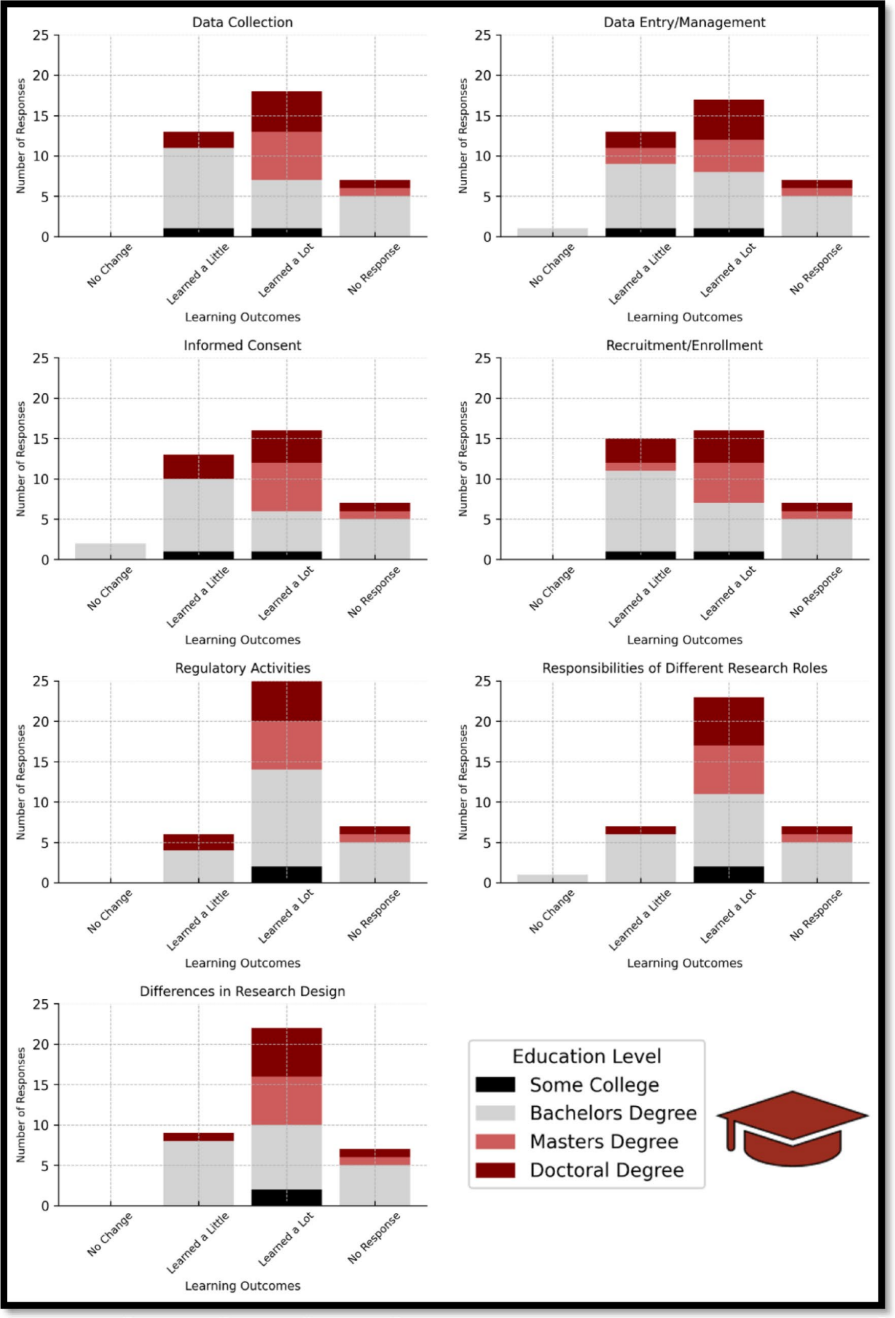
Learning outcomes by educational Level

**Fig. 3:**
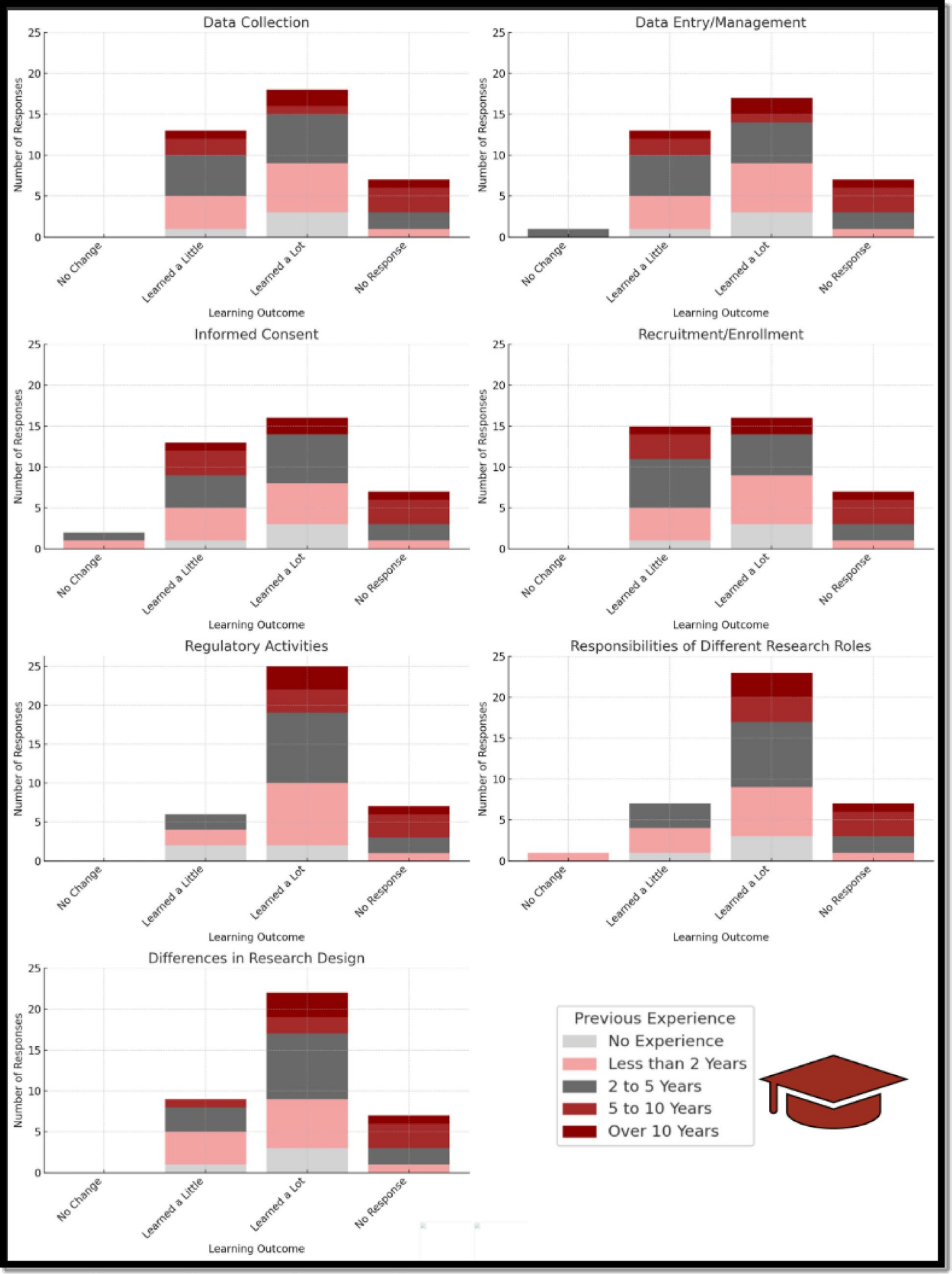
Learning outcomes by experiential Level

Stratified analysis of responses showed that participants with advanced degrees (master’s or doctoral) were more likely to report learning a lot in domains related to research design, regulatory processes, and data collection—areas in which they likely had prior academic exposure. Participants with bachelor’s degrees, by contrast, reported notable gains in understanding Regulatory Activities and Research Design, suggesting that the badge filled important foundational knowledge gaps. These findings support the badge’s ability to foster skill development across a range of educational levels and enhance readiness for clinical research coordinator roles (See Fig. 2 Learning Outcomes by Educational Level and Fig. 3 Learning Outcomes by Experiential Level)**.**

### Longitudinal Survey Participation

While 38 participants completed the immediate post-course survey, response rates for longitudinal follow-up surveys declined significantly. The six-month survey yielded seven responses, and the twelve-month survey received only three. Although early responses provided insights into short-term impacts—particularly related to onboarding and initial job performance—the low follow-up response rate limits the ability to draw definitive conclusions about the long-term educational or professional effects of the training. Future evaluations may benefit from integrating follow-up surveys into formal performance reviews or offering completion incentives.

### Professional Development Intentions

The post-course survey also assessed whether the CRC badge influenced participants’ future professional goals. More than half (55%) of respondents reported that the course introduced them to new areas of clinical research they wished to explore further. A total of 26% indicated an intention to take additional clinical research courses, and 52% reported plans to pursue further credentialing, such as certifications or advanced degrees. Only one participant reported no effect on their future plans. These results indicate that the CRC Badge was successful in sparking interest in continued education and advancement in clinical research careers.

### RCI Administrative Feedback

Administrative feedback collected at 3 and 6 months post-implementation reflected strong support for the CRC Badge as an onboarding tool. Rutgers Cancer Institute managers reported that newly hired staff who completed the badge reached job readiness more quickly, demonstrated improved understanding of key concepts, and required less time for training on foundational topics. Administrators noted that the badge enhanced performance in specific areas such as informed consent, serious adverse event reporting, and data management. Many CRC badged employees remained in their positions over the following year, with some having received promotions. As a result, Rutgers Cancer Institute formally integrated the CRC Badge into its standard onboarding procedures.

However, the use of the CRC badge for continuing education among existing staff was less successful. Of the 56 staff members enrolled, 18 did not complete the training, and this group consisted largely of existing employees. Administrators attributed non-completion to time constraints and workload demands that made it difficult for staff to engage with the 20-h course. Attempts to complete the modules in short intervals between work tasks reportedly hindered learning retention. This limitation led administrators to conclude that while the badge is highly effective for onboarding, its application for continuing education would require structural adjustments to better accommodate staff schedules.

Overall, the results strongly support the CRC Badge as an effective, scalable training strategy for onboarding clinical research staff, with potential to strengthen workforce readiness and foster long-term professional growth, particularly in resource-constrained institutional environments.

## Discussion: The Value and Limitations of Micro-credentialing in a Lean Economic Environment

In the context of declining indirect cost reimbursement rates, academic medical centers face mounting pressure to reduce operational expenses while maintaining high standards in clinical research training and oversight. This environment necessitates creative and scalable solutions to sustain workforce quality and regulatory compliance. The implementation of the CRC Badge at Rutgers Cancer Institute represents one such approach, a targeted micro-credential designed to provide foundational training in core clinical research competencies. The results of our initial evaluation suggest that micro-credentials may serve as a practical and cost-sensitive mechanism for professional development and workforce optimization, particularly when deployed strategically.

Survey data revealed that over 90% of badge participants reported learning either “a little” or “a lot” across all core content areas. A majority of users indicated that the badge experience inspired them to pursue additional educational opportunities in the clinical research field. This suggests that the CRC badge was not only effective for foundational knowledge acquisition but also fostered continued professional engagement which is a beneficial outcome considering institutions may need to increasingly rely on internal retention and advancement due to restricted indirect funding.

Feedback from Rutgers Cancer Institute administrators further underscored the operational value of the badge. Clinical research staff required less time to be trained on foundational topics, allowing institutional resources to be redirected toward site-specific onboarding and protocol instruction. This time savings reflects a key advantage of micro-credentialing: redistribution of training responsibility. By covering general competencies externally, institutions may reduce demands on senior staff and training infrastructure.

The implementation also revealed important limitations when the CRC badge was positioned solely as a continuing education activity. Staff already in defined roles, without a need for retraining or upskilling, struggled to complete the 20-h training within the six-week window. Competing clinical and administrative responsibilities limited their availability for non-mandatory learning, highlighting the challenge of embedding time-intensive training into already saturated work schedules. In these cases, the badge was perceived as burdensome rather than beneficial, and its effectiveness diminished. This underscores that the success of micro-credentialing in lean environments depends not only on content quality and scalability, but also on intentional integration into workforce development plans.

Despite this limitation, micro-credentials like the CRC Badge offer several strategic advantages when thoughtfully aligned with institutional needs. They can support recruitment by expanding the eligible applicant pool to include individuals from diverse or non-traditional backgrounds. They provide a mechanism for retraining existing staff to fill gaps left by hiring freezes or budget cuts. Their modular, asynchronous design makes them well suited for scalable, remote delivery. The structured, verifiable nature of the training supports compliance documentation and audit preparedness, particularly important when regulatory standards persist despite constrained infrastructure budgets.

Taken together, the implementation of the CRC Badge at Rutgers Cancer Institute illustrates that micro-credentialing can serve as an effective tool in workforce development and institutional resilience when deployed in the right context. It is most valuable when aligned with hiring, onboarding, or retraining goals, especially where financial constraints require efficiency. However, it may be less effective as a stand-alone continuing education mechanism for established staff without clear incentives or time allocation. These findings highlight the importance of strategic deployment, targeted use cases, and administrative support in realizing the full potential of badges in a lean economic environment.

## Data Availability

No datasets were generated or analysed during the current study.
